# Quantitative Profiling of WNT-3A Binding to All Human Frizzled Paralogues in HEK293 Cells by NanoBiT/BRET Assessments

**DOI:** 10.1021/acsptsci.1c00084

**Published:** 2021-05-11

**Authors:** Paweł Kozielewicz, Rawan Shekhani, Stefanie Moser, Carl-Fredrik Bowin, Janine Wesslowski, Gary Davidson, Gunnar Schulte

**Affiliations:** † Section of Receptor Biology & Signaling, Dept. Physiology & Pharmacology, Karolinska Institutet, S-17165, Stockholm, Sweden; ‡ Institute of Biological and Chemical Systems-Functional Molecular Systems (IBCS-FMS), Karlsruhe Institute of Technology (KIT), 76131, Karlsruhe, Germany

**Keywords:** WNT, Frizzled, bioluminescence resonance energy transfer (BRET), NanoBiT/BRET, ligand binding, G protein-coupled receptor (GPCR)

## Abstract

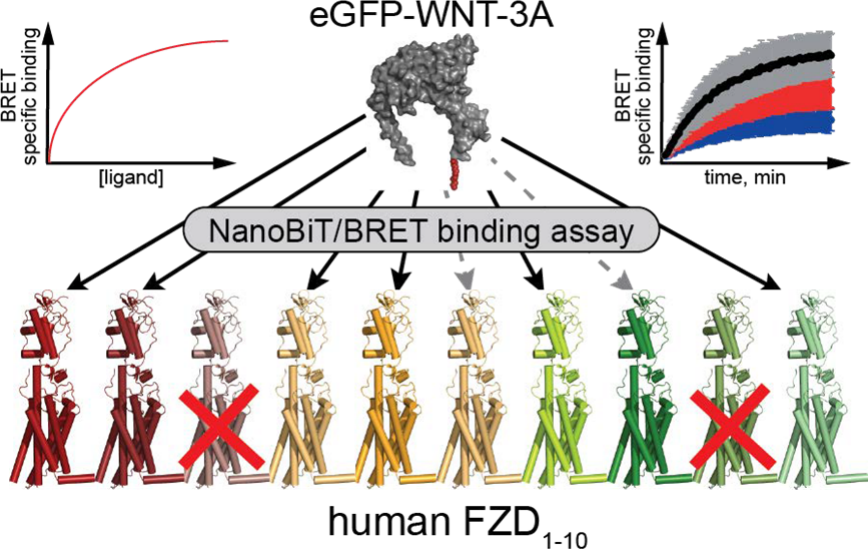

The WNT signaling system governs critical processes during embryonic development and tissue homeostasis, and its dysfunction can lead to cancer. Details concerning selectivity and differences in relative binding affinities of 19 mammalian WNTs to the cysteine-rich domain (CRD) of their receptors—the ten mammalian Frizzleds (FZDs)—remain unclear. Here, we used eGFP-tagged mouse WNT-3A for a systematic analysis of WNT interaction with every human FZD paralogue in HEK293A cells. Employing HiBiT-tagged full-length FZDs, we studied eGFP-WNT-3A binding kinetics, saturation binding, and competition binding with commercially available WNTs in live HEK293A cells using a NanoBiT/BRET-based assay. Further, we generated receptor chimeras to dissect the contribution of the transmembrane core to WNT-CRD binding. Our data pinpoint distinct WNT-FZD selectivity and shed light on the complex WNT-FZD binding mechanism. The methodological development described herein reveals yet unappreciated details of the complexity of WNT signaling and WNT-FZD interactions, providing further details with respect to WNT-FZD selectivity.

The ten mammalian Frizzleds (FZD_1–10_) are G protein-coupled receptors (GPCRs) and form—together with Smoothened (SMO)—the class F of GPCRs.^1,2^ The 19 different WNT lipoglycoproteins are the main macromolecular ligands of FZDs, interacting with the extracellular cysteine-rich domain (CRD) of the receptor. WNT-FZD signaling orchestrates multiple processes during embryonic development, stem cell regulation, and adult tissue homeostasis.^2^ Additionally, aberrant WNT signaling is implicated in tumorigenesis and other pathologies.^3,4^ Whereas recent advances have resulted in a better understanding of the underlying mechanisms controlling WNT-induced FZD activation and signal initiation, the relative binding affinities and ligand–receptor selectivity remain largely unknown.^5−13^ The quantitative assessment of WNT binding has been limited by the strong lipophilicity of WNTs, which makes their purification challenging and necessitates detergents and serum for solubilization and stabilization of WNTs, respectively.^14,15^ Nevertheless, WNT-FZD interactions were studied using biochemical and biophysical assays as well as through the use of *in silico* calculations.^16−22^ These studies generally reported WNT-FZD binding affinities in the range of 1–100 nM, which is reasonable when considering the known affinities of proteinaceous ligands to other GPCRs.^23,24^ Nevertheless, it remains unclear how these values translate into the physiological reality, since the local concentration of WNTs at the receptors *in vivo* remains unknown and is likely to be highly context-dependent.^25^ Until recently, the assessment of ligand binding was based on WNT binding to the CRD rather than the full-length FZD, or the reported assays were not performed in live cells. However, progress has been made with the use of FRET- and cpGFP-based biosensors to demonstrate WNT-induced FZD conformational dynamics and receptor activation,^7,12,26^ where WNT binding to a full-length receptor was reflected by an outward movement of the transmembrane domain TM6. Furthermore, the generation of a functional eGFP-tagged WNT-3A provided for the first time a biologically relevant FZD ligand that could be used as a probe in quantitative binding assays in real-time using living cells.^27,28^

Here, using live cell analysis of transiently transfected HEK293A cells overexpressing HiBiT-tagged FZDs, we provide a comparative assessment of binding affinities of eGFP-WNT-3A to all human FZD paralogues using kinetic and saturation binding formats. Furthermore, using a competition binding assay, we have assessed binding affinities of unlabeled, commercially available WNT proteins to FZD_4_. Finally, we have also explored the contribution of the FZD transmembrane core for the binding of WNTs to the primary FZD-CRD binding site.^29^ Compared with the previously described BRET-based assay for Nluc-FZD_4_ and Nluc-FZD_6_,^28^ we have used a nanoluciferase complementation-based BRET binding approach here, termed NanoBiT/BRET. In this BRET assay format, the fluorescent WNT-3A binds FZDs that are N-terminally tagged with the 11-amino-acid HiBiT peptide. The addition of the complementary LgBiT to the system allows rapid and high-affinity association to the HiBiT peptide, forming a stable NanoBiT moiety with a luciferase activity. This setup allows targeted analysis of cell surface receptors due to the cell impermeability of LgBiT, thereby providing a system with less intracellular background luminescence. This method is a modification of a well-established NanoBRET binding assay to study ligand–receptor association,^30^ and has been lately employed to study ligand binding to Class A GPCRs and receptor tyrosine kinases.^31−34^ Our results demonstrate that eGFP-WNT-3A interacts with full-length human FZDs transiently overexpressed in live HEK293A cells in a paralogue selective manner. This concept was expanded to unlabeled WNTs in competition binding experiments suggesting a complex WNT-FZD selectivity profile. The binding data based on full-length FZDs, FZD-CD86, and FZD-FZD chimeras underline the complexity of the WNT-FZD interaction and suggest that the core regions of FZDs may contribute to receptor selectivity.

## Results and Discussion

### eGFP-WNT-3A/FZD Binding Kinetics

**Figure 1:**
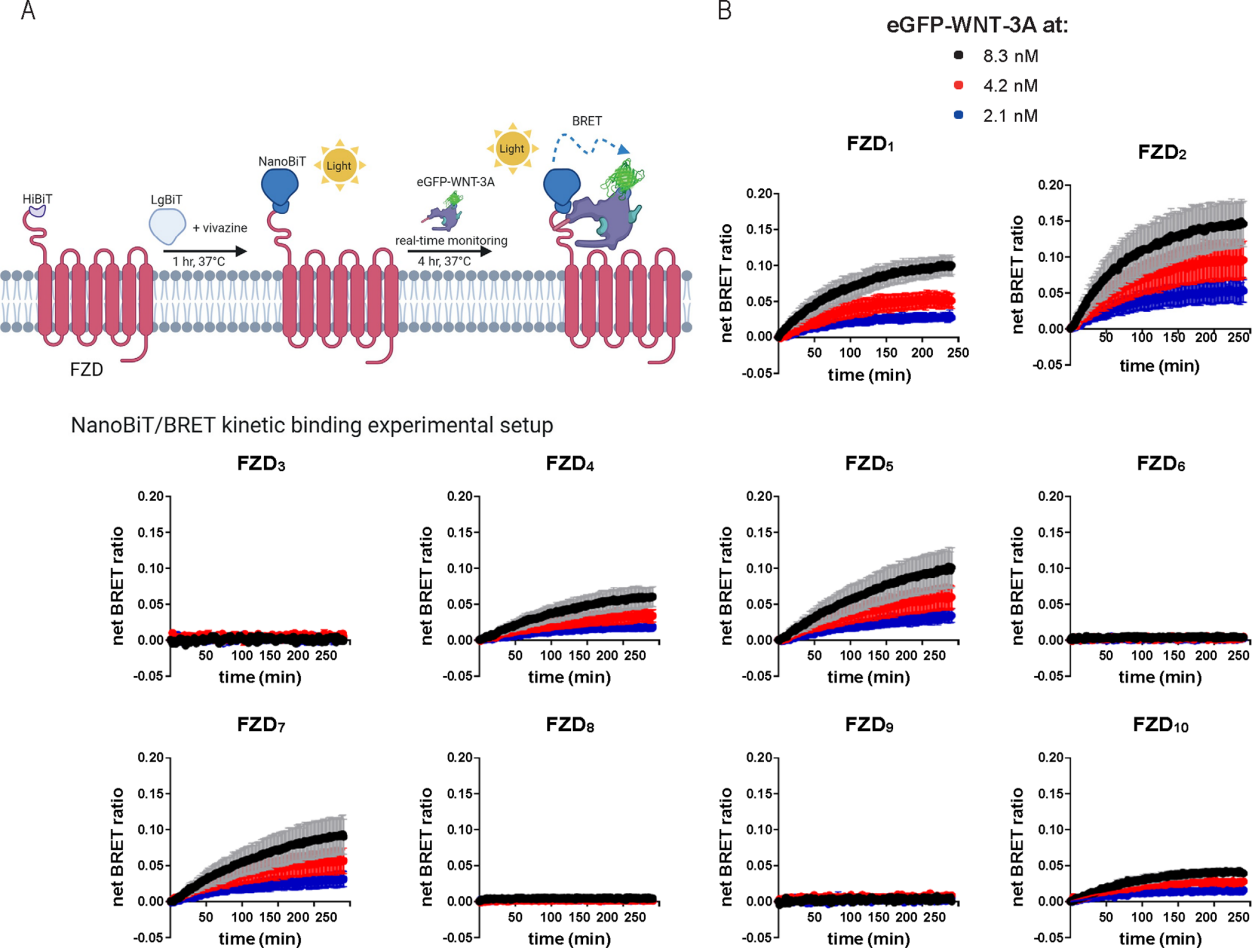
eGFP-WNT-3A binding kinetics. A. The scheme depicts the experimental setup of the NanoBiT/BRET analysis of association kinetics between the HiBiT-tagged FZD and the eGFP-WNT-3A. Created with BioRender.com. B. Association kinetics of the eGFP-WNT-3A to human HiBiT-FZDs were determined by the detection of NanoBiT/BRET in transiently overexpressing living HEK293A cells over time. BRET was sampled once per 90 s for 240 min. Data points are presented as means ± SEM from *n* = 3 individual experiments, fitting a two or more hot ligand concentrations kinetics model. Experiments were performed with eGFP-WNT-3A batch 1.

In order to establish a nanoluciferase complementation-dependent NanoBiT/BRET binding assay format to study all human FZD paralogues, we generated constructs for all 10 receptors carrying an N-terminal HiBiT tag (Figure S1A,B). Upon transient overexpression in HEK293A cells, all receptor constructs were detected at the cell surface, albeit with varying cell surface expression levels (Figure S1C). Additionally, HiBiT-tagged FZD_1_, FZD_2_, FZD_4_, FZD_5_, FZD_7_, FZD_8_, and FZD_10_ mediated WNT-3A-induced β-catenin-dependent signals as assessed by the TOPFlash reporter assay performed in HEK293T cells devoid of endogenous FZDs (ΔFZD_1–10_ HEK293T cells, Figure S1D(25)). In contrast, FZD_3_, FZD_6_, and FZD_9_ could not transduce WNT-3A-induced activation of this pathway, similar to what has been reported previously, yet with differing results for FZD_9_.^28,35−38^ Having verified the sequence and functionality of the HiBiT-tagged FZD constructs, HEK293A cells transiently overexpressing these FZDs were used in kinetic binding experiments. For these experiments, cells were first incubated with the complementary LgBiT protein and the luciferase substrate vivazine, and after 1 h, eGFP-WNT-3A was added to final concentrations of 2.1, 4.2, or 8.3 nM, and BRET readings were taken over a 4 h period at 37 °C (Figure 1A). The concentrations used for eGFP-WNT-3A were dictated by the maximal concentration that could be obtained for the eGFP-WNT-3A preparations and the assay format. We detected a saturable net BRET ratio indicative of eGFP-WNT-3A specific binding to HiBiT-tagged FZD_1_, FZD_2_, FZD_4_, FZD_5_, FZD_7_, and FZD_10_, with *K*_d_ values varying from 2.3 to 29.9 nM (Figure 1B, Table 1). In line with the TOPFlash data, no concentration-dependent increase in receptor–ligand BRET was detected for FZD_3_ and FZD_9_. Interestingly, FZD_8_, which maintained a strong WNT-3A-induced TOPFlash activity, and to a lesser extent FZD_6_, displayed very low but detectable binding that could be fitted to association curves over time (Figure S2A).

**Table 1:** Kinetic and Saturation Binding Parameters of eGFP-WNT-3A Binding to All 10 Human HiBiT-Tagged FZD Paralogues^a^

	FZD_1_	FZD_2_	FZD_3_	FZD_4_	FZD_5_	FZD_6_	FZD_7_	FZD_8_	FZD_9_	FZD_10_
Kinetic binding *K*_d_ (nM) ± SEM	29.9 ± 1.5	5.4 ± 0.1	n.d.	9.4 ± 0.5	2.3 ± 0.2	10.2 ± 3.7	2.8 ± 0.2	17.8 ± 4.4	n.d.	4.3 ± 0.3
Saturation binding *K*_d_ (nM) ± SEM	36.7 ± 12.7	48.6 ± 8.2	n.d.	17.7 ± 7.2	14.9 ± 7.6	6.5 ± 5.7	24.9 ± 9.9	4.9 ± 3.1	n.d.	21.3 ± 9.0

a *K*_d_ values are based on data from *n* = 3–4 individual experiments (shown in Figures 1B and 2B) and shown as a best-fit value ± SEM; n.d. = not determined.

### eGFP-WNT-3A/FZD Binding Affinity at Equilibrium

To define saturation binding affinity of eGFP-WNT-3A, we incubated human HiBiT-FZDs with a full concentration range (16.7 pM to 16.7 nM) of eGFP-WNT-3A for 240 min at 37 °C (Figure 2A). The net BRET ratio representing ligand–receptor binding increased in a clear, concentration-dependent manner for FZD_1_, FZD_2_, FZD_4_, FZD_5_, FZD_7_, and FZD_10_. Unfortunately, using transient overexpression of HiBiT-FZDs in HEK293A cells and the eGFP-WNT-3A with a limited maximal concentration in the conditioned medium, binding curves did not reach maximal asymptotic values but only came to near-saturable levels (Figure 2B). Again, detection of binding of eGFP-Wnt-3A to FZD_6_ and FZD_8_ was only marginally above background levels, as the net BRET values were low (Figure S2B). Similar to the kinetic binding assays, no quantifiable eGFP-WNT-3A binding was detected for FZD_3_ or FZD_9_. The affinities of eGFP-WNT-3A/FZD interactions were determined from linear regression curves showing near-saturable binding,^39^ and the *K*_d_ values are shown in Table 1. The reported saturation binding affinity values range from 4.9 to 48.6 nM, and they are in good agreement with the *K*_d_ values determined with kinetic binding for FZD_1_, FZD_4_, and FZD_6_. The degree of agreement is, however, only fair for FZD_5_, FZD_8_, and FZD_10_ and relatively poor for FZD_2_ and FZD_7_. Taken together, these kinetic and saturation binding data are in line with our previous results using fluorescence microscopy analysis, where no eGFP-WNT-3A association could be observed with C-terminally mCherry-tagged FZD_6_, and only a very weak association with FZD_8_ and FZD_9_ (FZD_3_ was not used).^28^ Although this fluorescence imaging-based method could provide an estimate of the relative ability of eGFP-WNT-3A to associate with different FZDs, accurate quantification of the binding affinities was not possible. Also, in that study, ΔFZD_1–10_ HEK293^GFP-free^ cells overexpressing FZD_8_-mCherry (but not FZD_6_-mCherry), showed very faint binding of eGFP-WNT-3A, and this is also in agreement with the HiBiT-tagged system used here, which can detect very low level, but specific, binding to FZD_8_ (Figure S2). This is in agreement with a recent report claiming that ligand–receptor interaction using the HiBiT-tagged system allows detection of very weak interactions.^32^ Furthermore, in the case of FZD_6_, there are differences in reports of its ability to bind or respond to WNT-3A. Biochemical experiments failed to detect any association between WNT-3A and FZD_6_-CRD-IgG,^16^ and no eGFP-WNT-3A/Nluc-FZD_6_ interaction was detected in our previous NanoBRET study.^28^ However, it should be noted that, compared with the HiBiT-tagged systems, binding analyses with Nluc-tagged receptors can display reduced sensitivity for detection of weak interactions, as recently discussed.^32^ On the other hand, recombinant human WNT-3A induced a conformational change in FZD_6_^12^ and affected the mobility of the receptor in the cell membrane as assessed by fluorescence recovery after photobleaching assay.^40^ Finally, the findings with respect to FZD_8_ are particularly intriguing, given the existing structural information on the WNT-3A-FZD_8_ CRD complex.^41^ It is currently unclear why such discrepancies exist for these two FZD paralogues.

**Figure 2:**
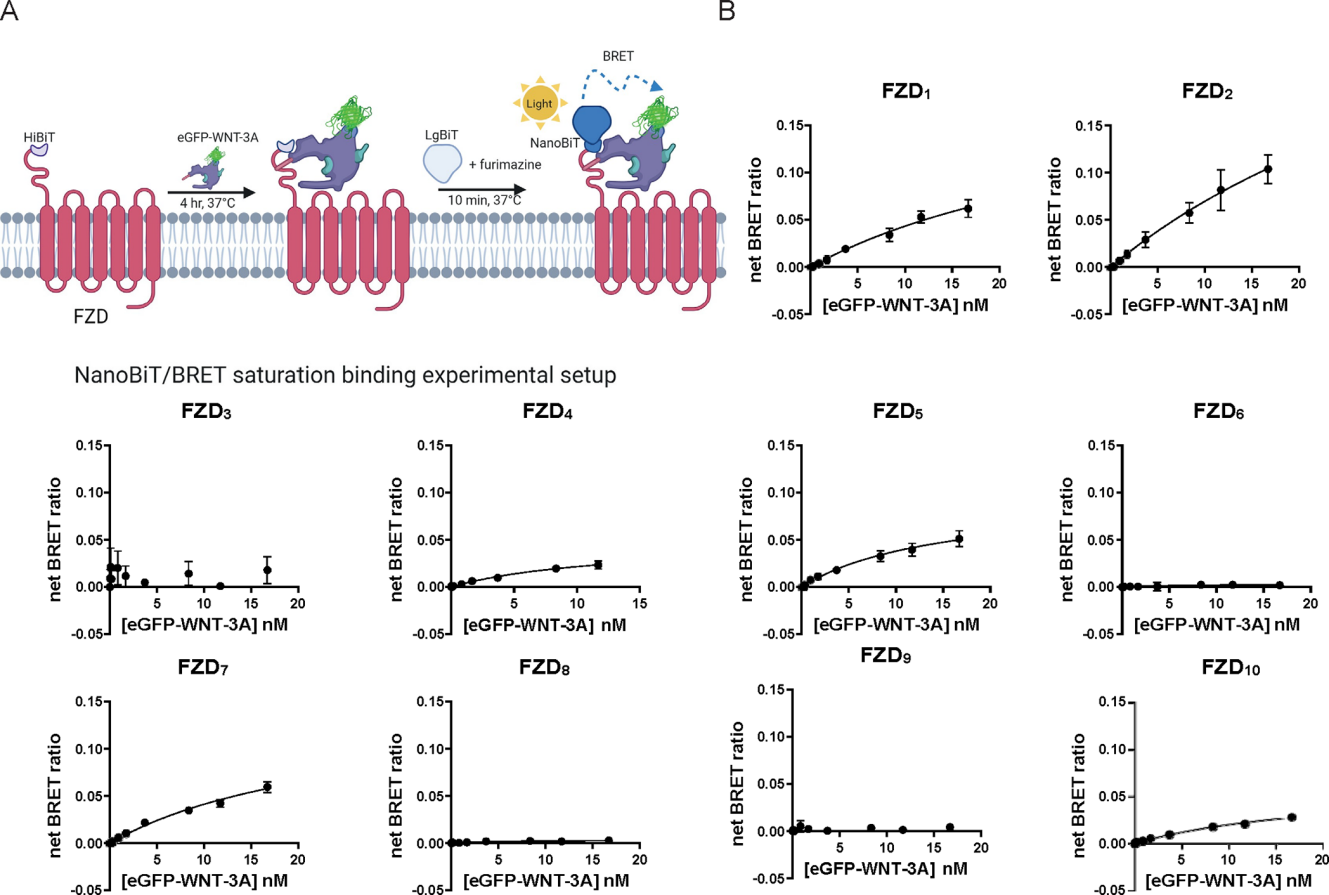
eGFP-WNT-3A saturation binding. A. The scheme depicts the experimental setup of NanoBiT/BRET analysis of equilibrium binding between the HiBiT-tagged FZD and the eGFP-WNT-3A. Created with BioRender.com. B. Saturation binding of the eGFP-WNT-3A to human HiBiT-FZDs was determined by the detection of NanoBiT/BRET in transiently overexpressing living HEK293A cells following 240 min incubation. Data points are presented as means ± SEM from *n* = 4 individual experiments, fitting a one-site specific model models. Experiments were performed with eGFP-WNT-3A batch 1.

**Table 2:** Binding Properties of Various FZD Ligands in Competition with eGFP-WNT-3A Binding (0.4 nM) to HiBiT-FZD_4_^a^

	WNT-3A	WNT-5A	WNT-5B	WNT-10B	WNT-11	WNT-16B
Competition binding p*K*_i_ ± SEM	7.26 ± 0.35	7.08 ± 0.21	6.93 ± 0.25	7.87 ± 0.55	8.90 ± 0.66	n.d.
ΔBRET ± SEM	–0.002 ± 0.001	–0.004 ± 0.0002	–0.004 ± 0.0002	–0.001 ± 0.0003	–0.001 ± 0.0004	n.d.
0.4 nM eGFP- WNT-3A binding displaced (%)	40.8	78.4	78.3	34.5	58.4	n.d.

a Data are based on *n* = 3–6 individual experiments presented in Figure 3B. p*K*_i_ values are presented as a best-fit value ± SEM; n.d. = not determined.

In order to directly compare NanoBiT/BRET and NanoBRET binding assay formats, we used Nluc-FZD_4_ and HiBiT-FZD_4_ constructs for saturation binding (Figure S3A–C). NanoBiT/BRET experiments were performed in two different experimental paradigms, where LgBiT protein was added either directly after (setup 1, used throughout this study Figure S3B and Figure 2A) or for 10 min before (setup 2, Figure S3C) the 4 h incubation with eGFP-WNT-3A. This allowed us to test for any potential steric hindrance of WNT binding to FZD caused by the presence or complementation of nanoluciferase. In these comparisons, although the differences in *K*_d_ values were apparent, they did not reach statistical significance (Nluc-FZD_4_*K*_d_ (nM) ± SEM = 7.6 ± 3.6; HiBiT-FZD_4_ setup 1 *K*_d_ (nM) ± SEM = 11.9 ± 3.6; HiBiT-FZD_4_ setup 2 *K*_d_ ± SEM (nM) = 20.4 ± 7.6). Furthermore, eGFP-WNT-3A binding to Nluc-FZD_4_ (Figure S3A) resulted in lower maximal BRET (BRET_max_) compared to either of the two HiBiT-FZD_4_ binding setups (Figure S3B,C) at similar luminescence levels (Nluc-FZD_4_ BRET_max_ ± SEM = 0.026 ± 0.006 vs HiBiT-FZD_4_ setup 1 BRET_max_ ± SEM = 0.049 ± 0.005, *P* = 0.0484; vs HiBiT-FZD_4_ setup 2 BRET_max_ ± SEM = 0.049 ± 0.004, *P* = 0.0349). This suggests that intracellular luminescence originating from receptors that are not accessible for the ligand reduces the assay’s dynamic range (see Figure S3D for expression analysis). In support of our choice to change from NanoBRET to the NanoBiT/BRET experimental setup, a recent study has shown that affinity measurements obtained from NanoBRET binding assays were generally less consistent in comparison with NanoBiT/BRET analyses.^32^

### eGFP-WNT-3A Competition Binding with Untagged WNTs at FZD_4_

With the aim to understand the competitive nature of eGFP-WNT-3A binding to FZD, we combined eGFP-WNT-3A with increasing concentrations of several commercially available and purified untagged WNT proteins: WNT-3A, WNT-5A, WNT-5B, WNT-10B, WNT-11, and WNT-16B. Again, we have used HiBiT-FZD_4_ as our model receptor. In this assay setup, HEK293A cells transiently overexpressing HiBiT-FZD_4_ were preincubated with the untagged WNTs for 30 min before addition of eGPF-WNT-3A to a final concentration of 0.4 nM. Cells were then incubated for a further 4 h to allow a competitive equilibrium to be reached before addition of LgBiT and subsequent BRET measurement (Figure 3A). The results of these experiments are shown in Figure 3B and summarized in the Table 2, and show that the untagged WNTs competitively displaced eGFP-WNT-3A from FZD_4_ with different affinities and capacities. Interestingly, WNT-10B and WNT-11 had the highest affinities, but they showed moderate BRET signal decrease as indicated by remaining residual BRET. Intriguingly, WNT-3A presented higher binding affinity (lower *K*_d_) but caused a lower reduction of BRET than WNT-5A and WNT-5B. WNT-16B did not compete with eGFP-WNT-3A in this FZD_4_-based assay. These results are in fair agreement with the recently published potencies and efficacies of WNTs in eliciting conformational changes in FZD_4_-cpGFP biosensor except for WNT-5B.^12^ Nevertheless, a similar rank order of affinities was obtained for WNT-3A, WNT-5A, and WNT-5B in binding to the isolated FZD_4_ CRD.^17^ Obviously, the insights into the mechanism of WNT–WNT competition for the primary binding site at FZDs remain obscure. We can only speculate that the process of functional ligand binding is more complex than for small-molecule ligands and other GPCRs. Along these lines, FZD oligomers associate or dissociate upon ligand addition,^42−44^ adding to the complexity of ligand binding analysis.^45^ Additionally, FZD coreceptors and various regulators could alter WNT interactions with FZD in HEK293 cells.^25^

**Figure 3:**
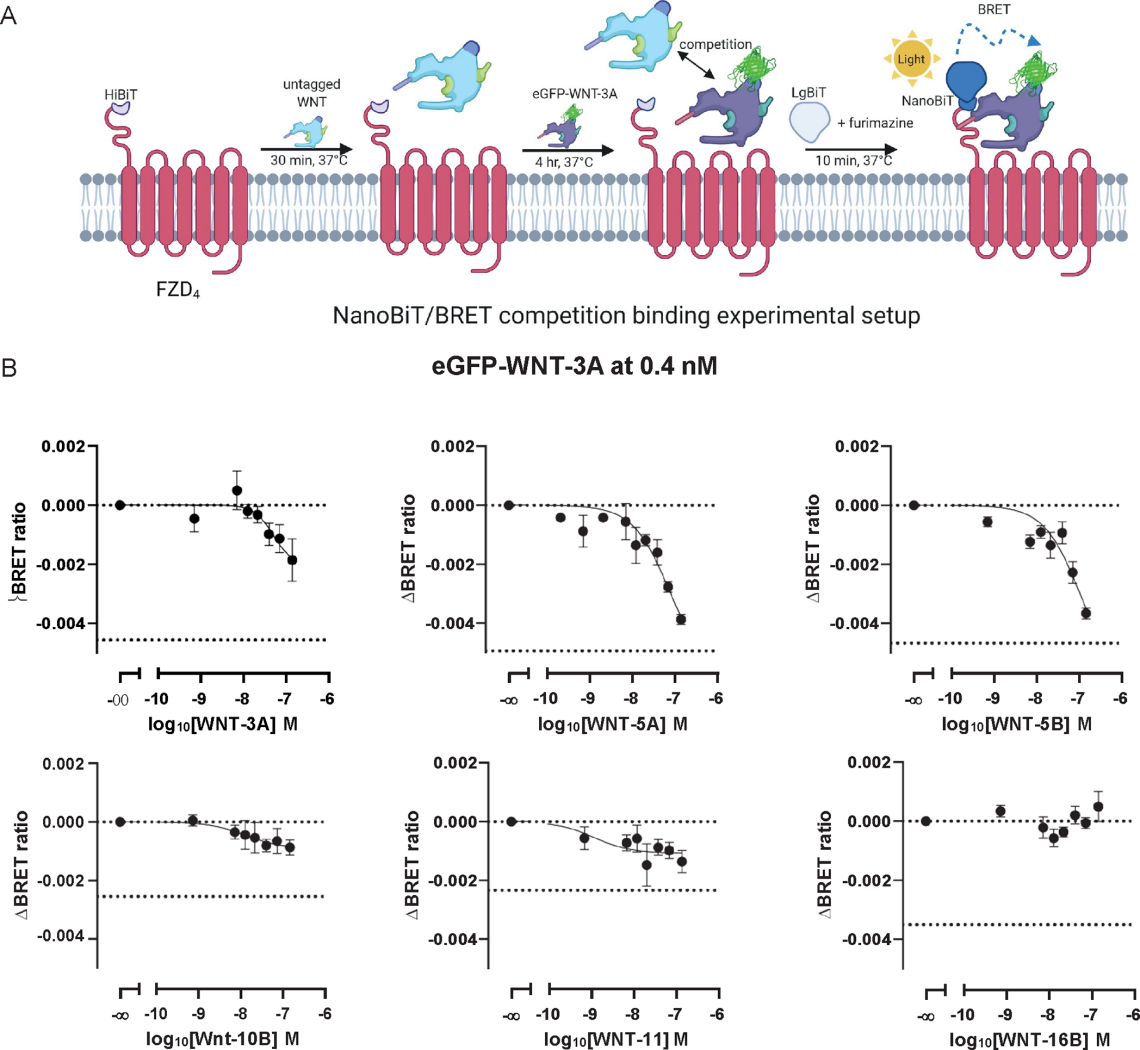
Competition binding between eGFP-WNT-3A and untagged WNTs at FZD_4_. A. The scheme depicts the experimental setup of NanoBiT/BRET analysis of competition binding between the eGFP-WNT-3A and commercially available untagged WNT-3A, WNT-5A, WNT-5B, WNT-10B, WNT-11, and WNT-16B. Created with BioRender.com. B. FZD_4_ binding of eGFP-WNT-3A at 0.4 nM in the presence of increasing concentrations of the untagged WNTs was determined by the detection of NanoBiT/BRET in transiently overexpressing living HEK293A cells following 240 min incubation. Data points are presented as means ± SEM from *n* = 3–6 individual experiments, fitting a three- or four-parameter model. Upper dashed line indicates the BRET ratio of eGFP-WNT-3A-only treated cells; lower dashed line indicates the BRET ratio of ligand-untreated cells (BRET donor only). Experiments were performed with eGFP-WNT-3A batch 2.

### Binding of eGFP-WNT-3A to FZD Chimeras

**Figure 4:**
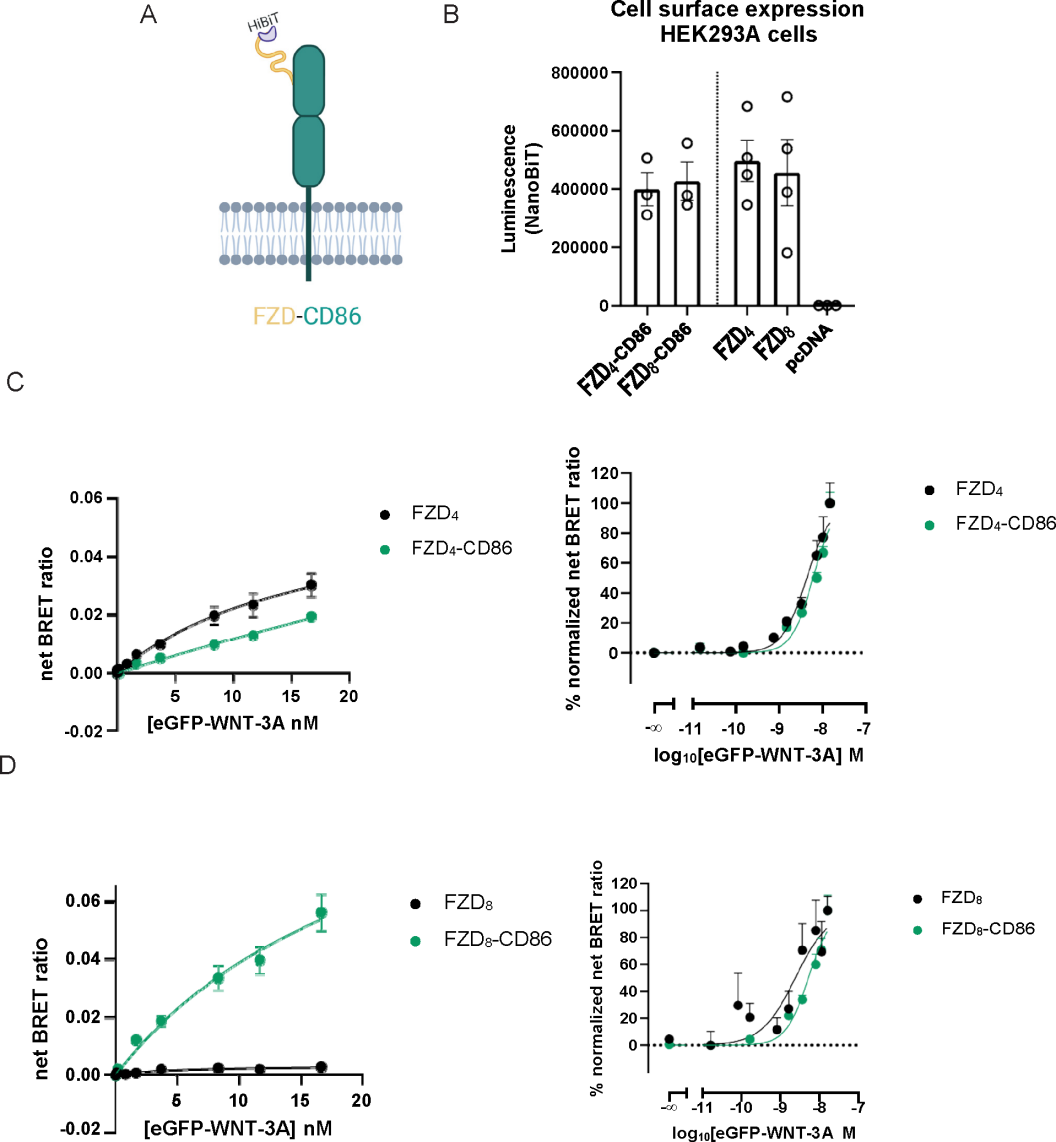
eGFP-WNT-3A binding to FZD-CD86 chimeras. A. The cartoon representations of the FZD-CD86 fusion proteins used in this study. eGFP-WNT-3A binding at equilibrium was assessed as depicted in Figure 2A. Created with BioRender.com. B. Cell surface expression of HiBiT-tagged FZD-CD86 chimeras as measured by NanoBiT luminescence (from the experiments summarized in Figure 2B and parts C–D). Data are presented as means ± SEM from *n* = 3–4 individual experiments. Expression data of FZD_4_, FZD_8_, and pcDNA are also depicted in SI Figure 1C. C. Saturation binding of eGFP-WNT-3A at FZD_4_-CD86 and FZD_4_ (data also present in Figure 2B) was determined by the detection of NanoBiT/BRET in transiently overexpressing living HEK293A cells following 240 min incubation. Data points are presented as means ± SEM from *n* = 3–4 individual experiments. eGFP-WNT-3A batch 1 was used in these experiments. D. Saturation binding of eGFP-WNT-3A at FZD_8_-CD86 and FZD_8_ (data also present in Figure 2B) was determined by the detection of NanoBiT/BRET in transiently overexpressing living HEK293A cells following 240 min incubation. Data points are presented as means ± SEM from *n* = 3–4 individual experiments. eGFP-WNT-3A batch 1 was used in the experiments. Linear scale data are fitted to a one-site specific binding model. Logarithmic-scale data are fitted to a normalized three- or four-parameter model. The right plot in every panel shows data normalized between 0% (BRET_min_) and 100% (BRET_max_) for each studied construct.

WNTs directly engage the CRD through protein–protein and protein–lipid interactions.^41,46^ However, it remains unclear whether non-CRD domains of FZDs contribute to WNT-FZD interaction.^20^ It has been hypothesized that the CRD simply serves the purpose of binding WNTs in order to bring them in close proximity with the receptor for additional binding/activation mechanisms.^47^ Indeed, the long and flexible nature of the linker connecting the CRD of FZDs to TM1 would tend to support such a hypothesis.^48^ The role of the FZD transmembrane domains in ligand binding, complex formation, receptor conformational changes, and signal transduction has been a subject of debate.^5,8,9,49−52^ Here, we seek to obtain a more mechanistic insight into the contribution of the transmembrane core to eGFP-WNT-3A binding. To this end, we generated two chimeric proteins fusing the N-terminal domain (NTD; CRD + linker) of one FZD with an unrelated CD86 single transmembrane domain spanning protein (Figure 4A). Specifically, we generated FZD_4_-CD86 and FZD_8_-CD86 chimeric proteins (Figure S4A). In this manner, we aimed to study the effect of a FZD core on eGFP-WNT-3A binding to the CRD. We validated the chimeras with regard to proper membrane trafficking upon transient overexpression in HEK293A cells and detected no difference in surface expression levels between FZD-CD86 and wild-type (WT) FZDs (Figure 4B).

In the NanoBiT/BRET binding experiments with the FZD_4_-CD86 chimera, we could detect a concentration-dependent increase in the BRET signal indicative of eGFP-WNT-3A binding. Interestingly, eGFP-WNT-3A interacted with FZD_4_-CD86 with a visibly lower affinity (higher *K*_d_) and a visibly lower maximal BRET (at the fixed concentrations used) than for an intact FZD_4_ protein (FZD_4_-CD86 *K*_d_ ± SEM (nM) = 141.9 ± 181.5, *P* = 0.0991; BRET_max_ ± SEM = 0.019 ± 0.001 vs FZD_4_ BRET_max_ ± SEM = 0.030 ± 0.004, *P* = 0.0622; Figure 4C). In order to emphasize differences in *K*_d_ for the tested receptors, the data were also normalized and are presented in a semilogarithmic presentation in Figure 4C (FZD_4_-CD86 p*K*_d_ ± SEM = 8.19 ± 0.04 vs FZD_4_ p*K*_d_ ± SEM = 8.31 ± 0.05, *P* = 0.0331). Importantly, differences in BRET_max_ for FZD_4_-CD86 and FZD_4_ cannot arise from differences in surface expression levels, as both studied receptors are similarly expressed (*P* = 0.3324).

Next, we performed a similar analysis for a FZD_8_-CD86 chimera. eGFP-WNT-3A binding to the chimera FZD_8_-CD86 compared to FZD_8_ at similar levels of receptor surface expressions (*P* = 0.8340; Figure 4B) did not differ in affinity in the analysis of non-normalized data (FZD_8_-CD86 *K*_d_ ± SEM (nM) = 22.9 ± 10.6, *P* = 0.6668; Figure 4D), but the difference reached statistical significance when comparing the normalized values (*P* = 0.0390; Figure 4D). Furthermore, the NanoBiT/BRET signal increased significantly when the FZD_8_ core was replaced by CD86. The NanoBiT/BRET signal (BRET_max_) was in fact comparable to other WNT-3A-binding competent FZDs (FZD_8_-CD86 BRET_max_ ± SEM = 0.056 ± 0.006 vs FZD_8_ BRET_max_ ± SEM = 0.003 ± 0.0003, *P* = 0.0002; Figure 4D and Figure 2). Intriguingly, these findings are the opposite of what we observe for FZD_4_, where replacing the receptor core with CD86 visibly reduced maximal BRET (Figure 4C). The efficiency of resonance energy transfer depends on both orientation and distance between BRET donor and BRET acceptor.^53^ Thus, in the NanoBiT/BRET binding setup, the differences in BRET_max_ can be interpreted as distinct ligand–receptor conformations. This further suggests that the cores of FZD_4_ and FZD_8_ can differently contribute to WNT-FZD binding.

In addition to the FZD-CD86 chimeras detailed above, we also generated FZD-FZD chimeras. Specifically, we constructed FZD_4_-FZD_6_, FZD_4_-FZD_8_, FZD_5_-FZD_6_, FZD_5_-FZD_7_, and FZD_6_-FZD_4_ chimeric proteins (Figure S4B). In this manner, we have used at least one FZD paralogue from every FZD homology cluster. The transmembrane cores of FZD_6_ and FZD_8_ were selected to test whether they negatively affect eGFP-WNT-3A binding to the CRD of FZD_4_ and FZD_5_. On the other hand, the FZD_7_ core was chosen to assess if it positively modulates ligand binding to the CRD of FZD_5_. The rationale for this selection was based on the very weak NanoBiT/BRET signal seen for eGFP-WNT-3A binding to FZD_6_ and FZD_8_, and strong NanoBiT/BRET signal of eGFP-WNT- 3A/FZD_7_ association in the two assay paradigms described in this study (Figure 1B and Figure 2B). Additionally, the FZD_6_ core was replaced with the FZD_4_ core to assess whether eGFP-WNT-3A binding to FZD_6_ CRD would increase upon insertion of a core from an eGFP-WNT-3A binding-competent FZD paralogue (Figure 1B and Figure 2B). We validated the chimeras with regard to their proper membrane trafficking upon transient overexpression in HEK293A cells and detected that the FZD-FZD chimera proteins are relatively poorly expressed on the cell surface compared to WT receptors (Figure S4C). Binding affinities of fluorescent propranolol to HiBiT-tagged β2-adrenergic receptors vary depending on the protein expression levels, with higher expression levels generally resulting in slightly elevated *K*_d_ values (lower affinity).^32^ Furthermore, binding affinities of DKK1-eGFP proteins to the WNT coreceptor LRP6-mCherry measured by dual-color axial line-scanning FCS (axial lsFCS) were higher for lower, more physiologically relevant receptor expression levels.^54^ Our data for transient overexpression of FZD_4_ in HEK293A cells mostly support these notions, with some exceptions (Figure S4D). Overall, it needs to be emphasized that although the *K*_d_ is a thermodynamic parameter that should be constant for various expression levels, differences in the cellular context may lead to different functionalities of overexpressed receptors present on the cell surface. This in turn can affect the comparative analysis and interpretation of real-time ligand binding data.^54^ However, provided the availability of cell surface expression data in the experimental paradigm of the NanoBiT/BRET binding assay, we shed light on a potential role of the receptor core for WNT-CRD binding. Focusing on the FZD_4_ chimeras, eGFP-WNT-3A bound with a significantly higher affinity (lower *K*_d_) to the FZD_4_-FZD_6_ and the FZD_4_-FZD_8_ chimeras compared to full-length FZD_4_ (FZD_4_-FZD_6_*K*_d_ ± SEM (nM) = 3.5 ± 1.8, *P* = 0.0255; FZD_4_-FZD_8_*K*_d_ ± SEM (nM) = 2.9 ± 1.3, *P* = 0.0371; Figure S4E). Interestingly, BRET_max_ values were visibly or significantly lower for both weakly expressed FZD_4_-FZD chimeras than for the intact FZD_4_ (FZD_4_-FZD_6_ BRET_max_ ± SEM = 0.021 ± 0.003, *P* = 0.0828; FZD_4_-FZD_8_ BRET_max_ ± SEM = 0.010 ± 0.001, *P* = 0.0192).

Additionally, our data showed that in comparison to WT FZD_5_, eGFP-WNT-3A binding to FZD_5_-FZD_6_ occurred with only visibly higher affinity, but this difference did not reach statistical significance (FZD_5_-FZD_6_*K*_d_ ± SEM (nM) = 4.4 ± 1.5, *P* = 0.0638; Figure S4F). Next, the affinity of eGFP-WNT-3A binding to FZD_5_-FZD_7_ was also not significantly different compared with WT FZD_5_ (FZD_5_-FZD_7_*K*_d_ ± SEM (nM) = 5.0 ± 2.4, *P* = 0.2622; Figure S4F). Moreover, the differences in BRET_max_ did not reach statistical significance (FZD_5_-FZD_6_ BRET_max_ ± SEM = 0.080 ± 0.014, *P* = 0.1715; FZD_5_-FZD_7_ BRET_max_ ± SEM = 0.098 ± 0.026, *P* = 0.2046; FZD_5_ BRET_max_ ± SEM = 0.051 ± 0.009).

In addition, our data indicated that eGFP-WNT-3A bound to FZD_6_-FZD_4_ with the same affinity (FZD_6_-FZD_4_*K*_d_ ± SEM (nM) = 5.8 ± 3.4, *P* = 0.9695; Figure S4G) as to FZD_6_ but with a significant, over 10-fold increase in the maximal BRET signal (FZD_6_-FZD_4_ BRET_max_ ± SEM = 0.030 ± 0.006 vs FZD_6_ BRET_max_ ± SEM = 0.002 ± 0.001, *P* = 0.056) arguing that the FZD_6_CRD can efficiently bind eGFP-WNT-3A in the context of a different receptor core. As mentioned before, it needs to be emphasized that receptor expression levels can affect ligand–receptor interaction. Along these lines, in classical BRET titration experiments, increasing BRET donor amounts (by increasing plasmid DNA amounts) with constant BRET acceptor levels (unchanged plasmid DNA amounts) leads to a decrease in BRET_max_ signal for specific donor–acceptor interactions.^55^ In contrast, no such relationship was found in our FZD-CD86 and FZD-FZD NanoBiT/BRET binding experiments, further supporting the notion that the FZD core has a differential role in WNT binding depending on the FZD paralogue.

Here, we have used the NanoBiT/BRET system to analyze the contribution of the FZD seven-transmembrane-spanning core to eGFP-WNT-3A binding to the CRD. We show that swapping the receptor core can have a substantial effect on the affinity or maximal BRET of eGFP-WNT-3A binding in the NanoBiT/BRET read-out. Our data, particularly from the FZD-CD86 experiments, argue that the seven-transmembrane-spanning core contributes to ligand binding for the tested FZDs, even though the details on the molecular level remain obscure.

This study adds substantial methodological advance to the pharmacological toolbox suitable for the study of the class F GPCRs and their coreceptors.^28,54,56^ We have demonstrated the vast potential of employing fluorescent WNTs and the NanoBiT/BRET binding technique for the pharmacological quantification of WNT-FZD interactions in live HEK293A cells despite the limitations that come with the low concentration of the tracer WNT in the conditioned medium preparation. The broader analysis of the selectivity of ligand–receptor interactions, WNT binding in the presence or absence of either FZD coreceptors, FZD-binding intracellular transducer proteins and at different FZD expression levels, can now be further investigated to understand the pluridimensionality of WNT-FZD system in a more physiologically relevant cell system.

## Experimental Section

### Cell Culture and Ligands

HEK293A cells (ATCC), HEK293F (Thermo Fisher Scientific, Waltham, MA, USA), HEK293T (DSMZ ACC-635), and ΔFZD_1–10_ HEK293T cells^25^ were cultured in DMEM supplemented with 10% FBS, 1% penicillin/streptomycin, and 1% l-glutamine (all from Thermo Fisher Scientific, Waltham, MA, USA) in a humidified CO_2_ incubator at 37 °C. All cell culture plastics were from Sarstedt (Nümbrecht, Germany), unless otherwise specified. The absence of mycoplasma contamination was routinely confirmed by PCR using 5′-GGCGAATGGGTGAGTAACACG-3′ and 5′-CGGATAACGCTTGCGACTATG-3′ primers detecting 16 S rRNA of mycoplasma in the media after 2–3 days of cell exposure. Untagged human WNT-3A, human/mouse WNT-5A, human WNT-5B, human WNT-10B, human WNT-11, and human WNT-16B were all from RnD Systems/Biotechne (#5036-WN, #645-WN, #7347-WN, #7196-WN, #6179-WN, and #7790-WN, Minneapolis, MI, USA). WNTs were dissolved at 100 μg/mL in filter-sterilized 0.1% BSA/PBS and stored at 4 °C. Molecular weights of the WNTs were as per supplier’s datasheets. Porcupine inhibitor C59 was from Abcam (#ab142216, Cambridge, UK).^57^ C59 was dissolved in DMSO at 5 mM and stored at −20 °C. The serial dilutions of WNTs were prepared in the protein-low binding tubes (Eppendorf, Hamburg, Germany).

### Preparation of eGFP-WNT-3A CM

HEK293F suspension cells growing in serum-free Expi293 expression medium (60 mL, 2.5 × 10^6^ cells/mL) were cotransfected with 10 μg of either *pCS2*^+^-*WNT-3A* or *pCS2*^+^-*eGFP-WNT- 3A* together with 50 μg of *pCMV-His-Afamin* plasmid using ScreenFect UP-293 (ScreenFect GmbH, Eggenstein-Leopoldshafen, Germany) according to the manufacturer’s instructions. The corresponding control CM was generated from cells transfected with *pCS2*^*+*^ plasmid.

Cells were first cleared from the HEK293F CM by centrifugation at 260 *g* (1200 rpm) for 10 min and then at 2800 *g* (4000 rpm) for 30 min to remove any remaining cellular debris and insoluble material. This “raw” CM then was concentrated 5-fold using Vivaspin turbo 15 centrifugal concentrators (30,000-molecular-weight-cutoff, Satorius AG, Göttingen, Germany) and exchanged to the desired cell culture medium using Sephadex G-25 PD10 desalting columns (GE Healthcare Bio-Science, Freiburg, Germany). The final concentration and integrity of eGFP-WNT-3A in the CM samples were determined using ELISA (GFP ELISA kit, #ab171581, Abcam) and SDS-PAGE/Western Blot analysis, respectively. Two eGFP-WNT-3A batches (eGFP-WNT-3A batch 1 final concentration: 16.7 nM; eGFP-WNT-3A batch 2 final concentration: 16.2 nM) were used in this study. Current WNT purification methods allow only limited WNT concentration to be obtained from CM.^58^ For validation of the eGFP-WNT-3A batches, please see Figure S5.

### Plasmids

Generation of HiBiT-FZD_4_, HiBiT-FZD_6_, and Nluc-FZD_4_ has been described previously.^28^ Gibson cloning was used to generate other HiBiT-tagged receptor constructs using HiBiT-tagged backbone from HiBiT-FZD_4_ containing a 5-HT_3_A signal sequence. To generate chimeras, the N-terminal domains (NTD; CRD with a linker region) and the transmembrane cores were defined according to Frizzled structures predicted on GPCRdb (http://www.gpcrdb.org), and the constructs were generated with Gibson cloning. Nluc-CD86 used to generate FZD-CD86 chimeras was from Martin J. Lohse (Max-Delbrueck Center for Molecular Medicine, Berlin, Germany). Frizzled and CD86 signal peptides were defined with SignalIP-5.0 Server (http://www.cbs.dtu.dk/services/SignalP/). The constructs were validated by sequencing (Eurofins GATC, Konstanz, Germany). The details of the constructs used in this study are presented in Figure S1A,B and Figure S4A.

### NanoBiT/BRET Binding

HEK293A cells were transiently transfected in suspension using Lipofectamine 2000 (Thermo Fisher Scientific, Waltham, MA, USA). A total of 4 × 10^5^ cells were transfected in 1 mL with 1000 ng of HiBiT-tagged FZDs or 10 ng of Nluc-FZD_4_ plasmid DNA. The cells (50 μL) were seeded onto a poly(d-lysine)-coated black 96-well cell culture plate with a solid flat bottom (Greiner BioOne). Next, 50 μL of complete DMEM medium was added to each well. Forty-eight hours post-transfection, the cells were washed once with 200 μL of Hanks’ balanced salt solution (HBSS; HyClone). In the kinetic binding experiments, the cells were preincubated with 50 μL of a mix of Nluc substrate vivazine (1:50 dilution; #N2581, Promega, Fitchburg, WI, USA) and LgBiT (1:100 dilution; #N2421, Promega, Fitchburg, WI, USA) in a complete, nonphenol red DMEM (HyClone) supplemented with 10 mm HEPES for 1 h at 37 °C without CO_2_. Subsequently, 50 μL of eGFP-WNT-3A conditioned medium or control medium supplemented with 5% FBS and 10 mm HEPES was added, and the BRET signal was measured every 90 s for 240 min at 37 °C (161 measurements, no CO_2_). In the saturation-binding experiments, the cells were incubated with different concentrations of eGFP-WNT-3A conditioned medium (90 μL) supplemented with 5% FBS and 10 mm HEPES for 240 min at 37 °C with no CO_2_. In the competition binding experiments, the cells were preincubated for 30 min at 37 °C with 80 μL of unlabeled WNT proteins at 37 °C with no CO_2_. Subsequently, 10 μL of eGFP-WNT-3A at a concentration of 3.6 nM (final concentration of 0.4 nM) were added, and the cells were incubated for further 240 min at 37 °C with no CO_2_. Next, for saturation and competition binding experiments, 10 μL of a mix of furimazine (1:10 dilution; #N2421, Promega, Fitchburg, WI, USA) and LgBiT (1:20 dilution; #N2421, Promega, Fitchburg, WI, USA) was added. For saturation binding experiments with Nluc-FZD_4_ furimazine was used at 1:1000 final dilution (#N1572, Promega, Fitchburg, WI, USA) and no LgBiT was added. The cells were incubated for another 10 min at 37 °C with no CO_2_ before the BRET measurements. The BRET ratio was determined as the ratio of light emitted by eGFP (energy acceptor) and light emitted by HiBiT-FZD_1–10_ or Nluc-FZD_4_ (energy donors). The net BRET ratio was calculated as the difference in BRET ratio between cells treated with eGFP-WNT-3A, and cells treated with vehicle. ΔBRET ratio in the competition binding experiment was calculated as the difference in BRET ratio of cells treated with vehicle (eGFP-WNT-3A only wells, no ΔBRET) and cells treated with WNTs. The BRET acceptor (bandpass filter, 535–30 nm) and BRET donor (bandpass filter, 475–30 nm) emission signals were measured using a CLARIOstar microplate reader (BMG, Ortenberg, Germany). Cell surface expression of HiBiT-tagged FZDs and total expression of Nluc-FZD_4_ was assessed by measuring luminescence of vehicle-treated wells (no BRET acceptor) in the NanoBiT/BRET or NanoBRET binding assays, respectively. eGFP fluorescence was measured prior to reading BRET (excitation, 470–15 nm; emission, 515–20 nm).

### TOPFlash Reporter Gene Assay

ΔFZD_1–10_ HEK 293T cells were transfected in suspension (4 × 10^5^ cells were transfected in 1 mL) with 700 ng of HiBiT-tagged receptor, 250 ng M50 Super 8× TOPFlash (#12456; Addgene, Watertown, MA, USA), and 50 ng pRL-TK Luc (#E2241, Promega, Fitchurg, WI, USA) and seeded (50 μL) onto a poly(d-lysine)-coated white 96-well cell culture plate with a solid flat bottom (Greiner BioOne). Next, 50 μL of complete DMEM medium was added to each well. Twenty-four hours after transfection, the medium was changed to starvation medium (DMEM without FBS) containing either 8.0 nM (300 ng/mL) WNT-3A or vehicle, and 10 nM C59. Twenty-four hours after stimulation, cells were lysed gently shaking with 20 μL 1× Passive Lysis Buffer (#E1910; Promega, Fitchurg, WI, USA) for 15 min. Subsequently, 20 μL of LAR II (Promega, E1910) was added to all wells after which luminescence (580–80 nm) was read, and then 20 μL of Stop & Glo (Promega, E1910) was added to all wells after which luminescence (480–80 nm) was read again with a CLARIOstar microplate reader (BMG, Ortenberg, Germany).

### Data Analysis and Statistics

All data were analyzed in GraphPad Prism 8 (San Diego, CA, USA) using built-in equations. All data presented in this study come from *n* individual experiments (at least three biological replicates) with each individual experiment performed typically in duplicates (technical replicates) for each tested concentration/condition. Data points on the binding curves represent mean ± SEM. Saturation binding curves were fit using one-site-specific or total and nonspecific saturation nonlinear regression models (linear scale for eGFP-WNT-3A concentrations) or normalized three-parameter or normalized four-parameter nonlinear regression models (logarithmic scale for eGFP-WNT-3A concentrations with normalized net BRET ratio). The fitting models were selected based on an extra-sum-of-squares *F*-test (*P* < 0.05). Kinetic binding data were analyzed using the association model with two or more hot ligand concentrations. Binding affinity values (*K*_d_) are presented as a best-fit *K*_d_ with SEM. *K*_d_ values were compared using an extra-sum-of-squares *F*-test (*P* < 0.05). Competition binding curves were analyzed using a three- or four-parameter nonlinear regression model to obtain equilibrium dissociation constant values p*K*_i_ with SEM of unlabeled ligands as per the Cheng–Prusoff equation.^59^ Minimal BRET (BRET_min_) and maximal BRET (BRET_max_) were defined as the lowest and highest measured net BRET ratios, respectively. BRET_max_ values were compared using unpaired *t*-test. TOPFlash and cell surface expression data are presented as mean ± SEM. TOPFlash and cell surface expression data were analyzed for differences with Brown–Forsythe and Welch one-way analysis of variance (ANOVA); ** *P* ≤ 0.01, * *P* ≤ 0.05.

## Supporting Information Available

Detailed plasmid information, cell surface expression data, TOPFlash data, NanoBRET vs NanoBiT/BRET binding comparison data, detailed information about FZD-CD86 and FZD-FZD chimeras, eGFP-WNT-3A binding to FZD-FZD chimeras and eGFP-WNT-3A validation PDF)

## Abbreviations

CM: conditioned medium
CRD: cysteine-rich domain
eGFP: enhanced green fluorescent protein
FRET: Förster resonance energy transfer
FZD: Frizzled
GPCR: G protein-coupled receptor
HBSS: Hank’s balanced salt solution
NanoBiT/BRET: nanoluciferase binary technology/bioluminescence resonance energy transfer
Nluc: nanoluciferase
NTD: N-terminal domain
TCF/LEF: T-cell factor/lymphoid enhancer-binding factor
WNT: WNT/Int-1 family of proteins
WT: wild-type
