# Supplementary material for: Quantitative Profiling of WNT-3A Binding to All Human Frizzled Paralogues in HEK293 Cells by NanoBiT/BRET Assessments
Source: ACS Pharmacol Transl Sci. 2021 May 11;4(3):1235–45. doi: 10.1021/acsptsci.1c00084 (PMC8205236; doi:10.1021/acsptsci.1c00084)
Supplement: Supplementary file 1 — pt1c00084_si_001.pdf [file pt1c00084_si_001.pdf]

Supporting Information

Quantitative Profiling of WNT-3A Binding to All Human Frizzled Paralogues in HEK293 Cells by NanoBiT/BRET Assessments

Paweł Kozielewicz, Rawan Shekhani, Stefanie Moser, Carl-Fredrik Bowin, Janine Wesslowski, Gary Davidson* and Gunnar Schulte**

Corresponding Authors:

Paweł Kozielewicz - Section of Receptor Biology & Signaling, Dept. Physiology & Pharmacology, Karolinska Institutet, S-17165, Stockholm, Sweden; pawel.kozielewicz@ki.se

Gary Davidson – Institute of Biological and Chemical Systems-Functional Molecular Systems (IBCS-FMS), Karlsruhe Institute of Technology (KIT), Karlsruhe, Germany; gary.davidson@kit.edu

Gunnar Schulte - Section of Receptor Biology & Signaling, Dept. Physiology & Pharmacology, Karolinska Institutet, S-17165, Stockholm, Sweden; gunnar.schulte@ki.se

Authors:

Rawan Shekhani – Section of Receptor Biology & Signaling, Dept. Physiology & Pharmacology, Karolinska Institutet, S-17165, Stockholm, Sweden

Stefanie Moser – Institute of Biological and Chemical Systems-Functional Molecular Systems (IBCS-FMS), Karlsruhe Institute of Technology (KIT), Karlsruhe, Germany

Carl-Fredrik Bowin – Section of Receptor Biology & Signaling, Dept. Physiology & Pharmacology, Karolinska Institutet, S-17165, Stockholm, Sweden

Janine Wesslowski – Institute of Biological and Chemical Systems-Functional Molecular Systems (IBCS-FMS), Karlsruhe Institute of Technology (KIT), Karlsruhe, Germany

Table of content:

Supplementary Figure 1. Characteristics of HiBiT-FZD constructs used in this study

Supplementary Figure 2. eGFP-WNT-3A binding to FZD_6 and FZD_8 with enlarged scale

Supplementary Figure 3. eGFP-WNT-3A binding comparison between Nluc-FZD_4 and HiBiT-FZD_4

Supplementary Figure 4. Construct validation of FZD-FZD and FZD-CD86 fusion proteins. Analysis of correlation between receptor expression levels and ligand affinity. eGFP-WNT-3A saturation binding to FZD-FZD chimeras.

Supplementary Figure 5. Validation of eGFP-WNT-3A conditioned media (CM)

A

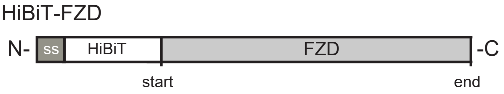

B

plasmid	source of ORF	FZD start	FZD end
HiBiT-FZD$_1$	synthetic from Twist Bioscience	Q73	V647
HiBiT-FZD$_2$	synthetic from IDT	F24	V565
HiBiT-FZD$_3$	mutated from mouse FZD$_3$-GFP (Jeremy Nathans)	H23	A666
HiBiT-FZD$_4$	FZD$_4$-Cerulean [1]	F37	V537
HiBiT-FZD$_5$	synthetic HA-FZD$_5$ (Thomas Sakmar)	A27	V585
HiBiT-FZD$_6$	SNAP-FZD$_6$ (Madelon Maurice)	H19	T706
HiBiT-FZD$_7$	SNAP-FZD$_7$ (Heptares)	Q33	V574
HiBiT-FZD$_8$	synthetic from IDT	A28	V694
HiBiT-FZD$_9$	FZD$_9$-GFP (OriGene)	L23	L591
HiBiT-FZD$_{10}$	FZD$_{10}$-GFP [2]	I21	V581

C

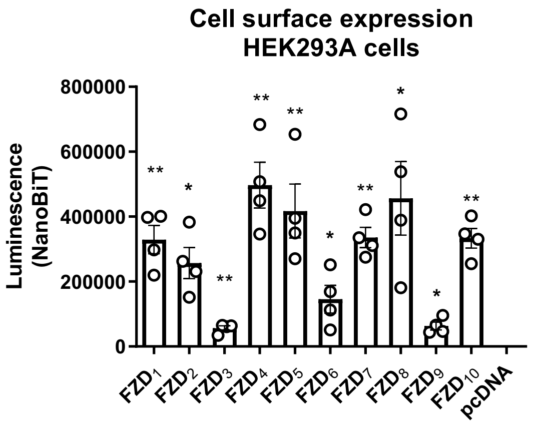

D

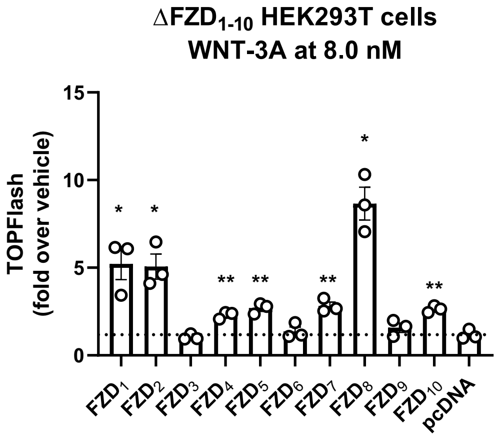

Fig. S1

Supplementary Figure 1. Characteristics of HiBiT-FZD constructs used in this study. A. Schematic representation of HiBiT-FZDs used to study eGFP-WNT-3A binding. ss – signal sequence. **B.** Source of receptor plasmid cDNA used to generate the N-terminally HiBiT-tagged constructs. First and last amino acids of each Frizzled protein are shown. **C.** Cell surface expression of HiBiT-FZDs as measured by NanoBiT luminescence (from the experiments summarised in **Figure 2B**). Data are presented as means ± SEM from n = 4 individual experiments. **D.** TOPFlash reporter activity of transiently overexpressed HiBiT-$FZD_{1\text{-}10}$ in $\Delta FZD_{1\text{-}10}$ HEK293T cells was assessed following 24hr stimulation with commercially-available untagged WNT-3A (8.0 nM) in the presence of 10 nM C59 in the serum-free conditions. Data are presented as means ± SEM from n = 3 individual experiments. TOPFlash and cell surface expression data were analyzed for differences with Brown-Forsythe and Welch one-way analysis of variance (ANOVA); ** $P \leq 0.01$, * $P \leq 0.05$.

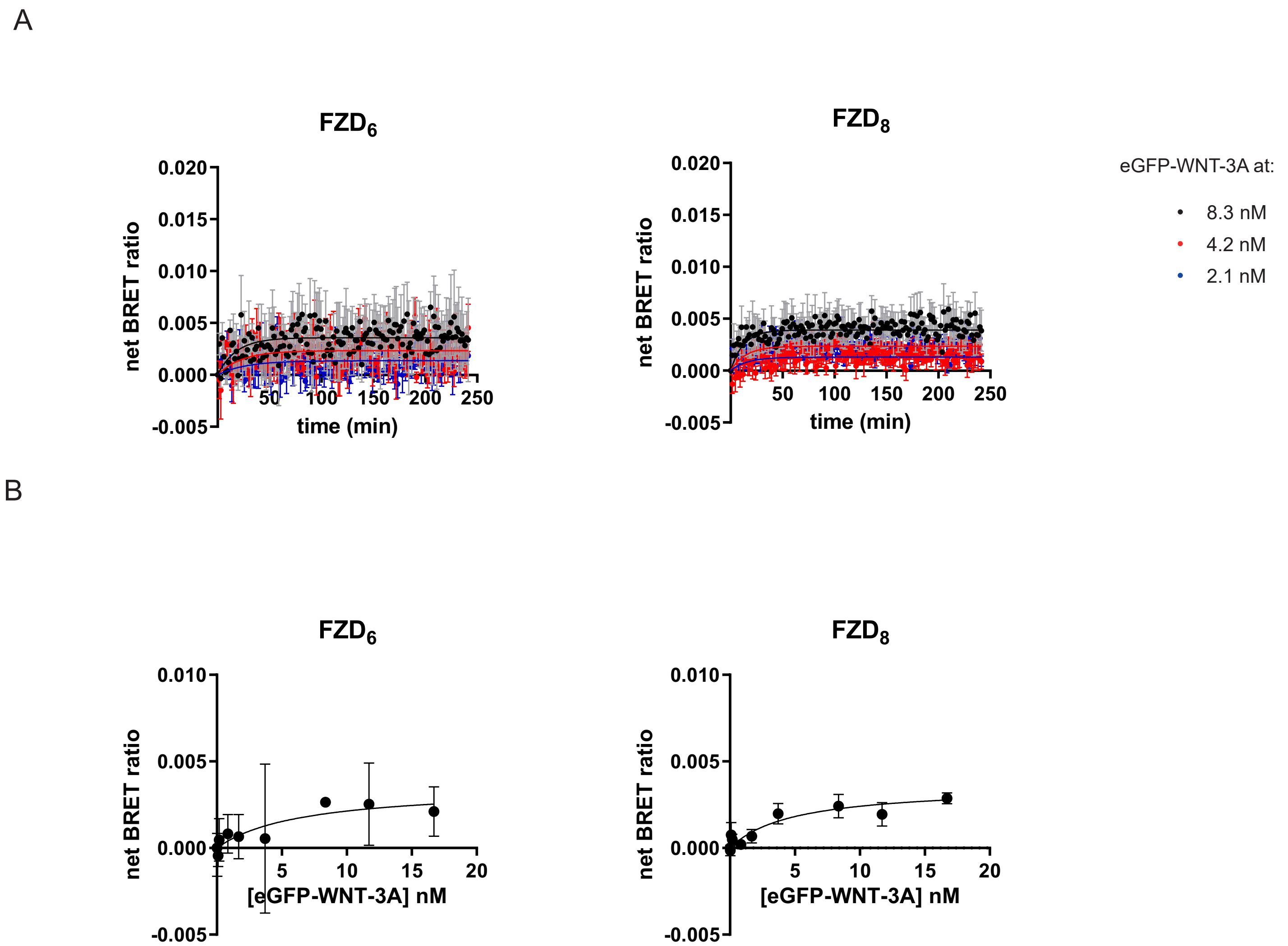
A
FZD6
0.020
0.015
0.010
0.005
0.000
-0.005
net BRET ratio
50
100
150
200
250
time (min)
FZD8
eGFP-WNT-3A at:
8.3 nM
4.2 nM
2.1 nM
B
FZD6
0.010
0.005
0.000
-0.005
net BRET ratio
5
10
15
20
[eGFP-WNT-3A] nM
FZD8

Fig. S2

Supplementary Figure 2. eGFP-WNT-3A binding to FZD_6 and FZD_8 A. Plots with association binding to FZD_6 and FZD_8 from **Figure 1** are presented with the enlarged scale on the y-axis to confirm weak but detectable NanoBiT/BRET signal. **B.** Plots with saturation binding to FZD_6 and FZD_8 from **Figure 2** are presented with the enlarged scale on the y-axis to confirm weak but detectable NanoBiT/BRET signal.

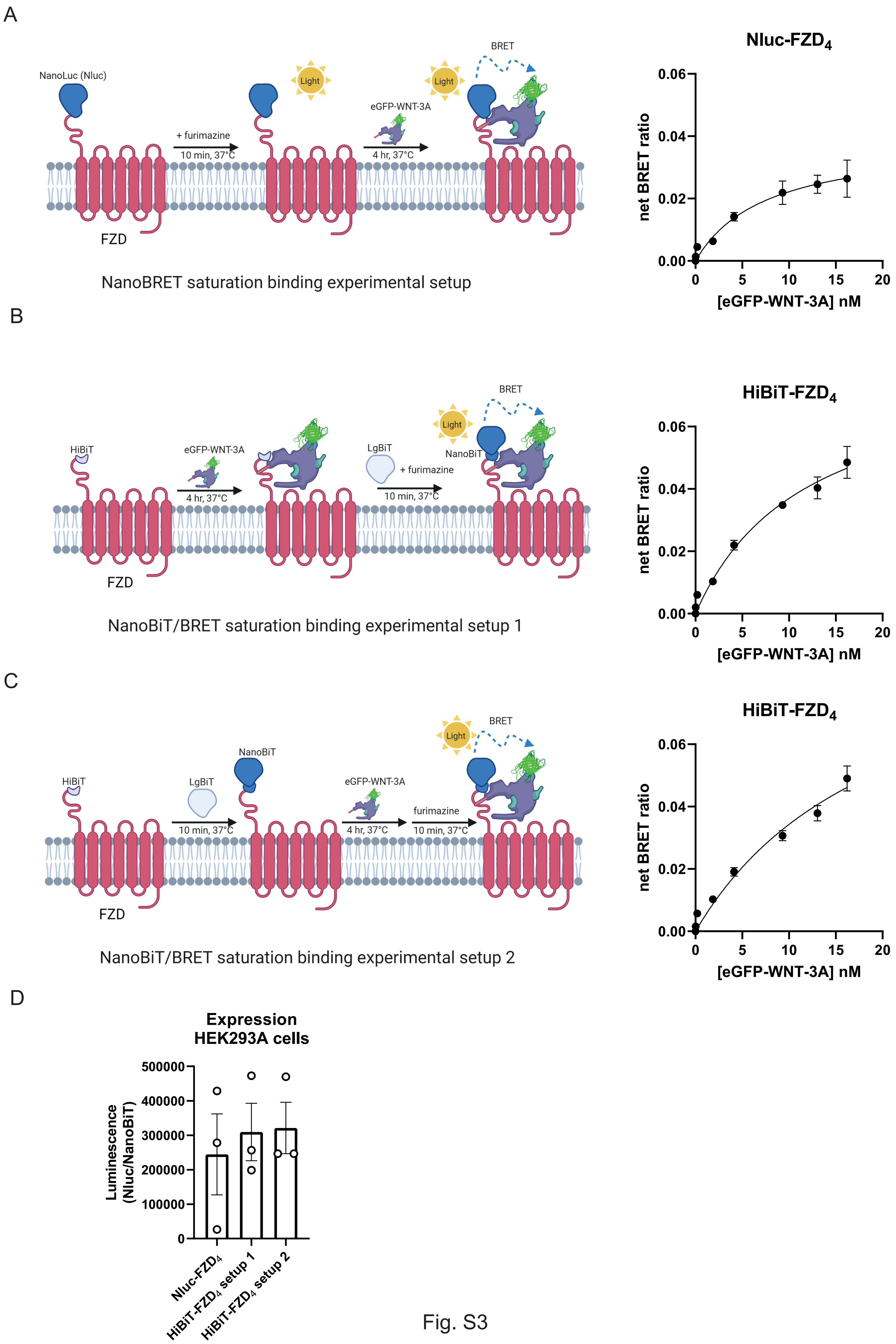
A
NanoLuc (Nluc)
+ furimazine
10 min, 37°C
Light
eGFP-WNT-3A
4 hr, 37°C
BRET
FZD
NanoBRET saturation binding experimental setup
Nluc-FZD4
net BRET ratio
0.00
0.02
0.04
0.06
0
5
10
15
20
[eGFP-WNT-3A] nM
B
HiBiT
eGFP-WNT-3A
4 hr, 37°C
LgBiT
+ furimazine
10 min, 37°C
NanoBiT
Light
BRET
FZD
NanoBiT/BRET saturation binding experimental setup 1
HiBiT-FZD4
net BRET ratio
[eGFP-WNT-3A] nM
C
HiBiT
LgBiT
10 min, 37°C
NanoBiT
eGFP-WNT-3A
4 hr, 37°C
furimazine
10 min, 37°C
Light
BRET
FZD
NanoBiT/BRET saturation binding experimental setup 2
HiBiT-FZD4
net BRET ratio
[eGFP-WNT-3A] nM
D
Expression
HEK293A cells
Luminescence
(Nluc/NanoBiT)
0
100000
200000
300000
400000
500000
Nluc-FZD4
HiBiT-FZD4 setup 1
HiBiT-FZD4 setup 2

Fig. S3

Supplementary Figure 3. eGFP-WNT-3A binding affinity to Nluc-FZD$_4$ and HiBiT-FZD$_4$ is similar. A. Left. The scheme depicts the experimental set up of NanoBRET analysis of equilibrium binding between the Nluc-FZD$_4$ and the eGFP-WNT-3A. Created with BioRender.com. **Right.** Saturation binding of the eGFP-WNT-3A to human Nluc-FZD$_4$ was determined by the detection of NanoBRET in transiently overexpressing living HEK293A cells following 240 min incubation. Data points are presented as means ± SEM from n = 3 individual experiments, fitting a one-site specific binding model. **B. Left.** The scheme depicts the experimental set up 1 of NanoBiT/BRET analysis of equilibrium binding between the HiBiT-FZD$_4$ and the eGFP-WNT-3A as used in the saturation binding experiments throughout this study. LgBiT and furimazine were added after 240 min incubation with the fluorescent probe. Created with BioRender.com. **Right.** Saturation binding of the eGFP-WNT-3A to human Nluc-FZD$_4$ was determined by the detection of NanoBRET in transiently overexpressing living HEK293A cells following the 240 min incubation. Data points are presented as means ± SEM from n = 3 individual experiments, fitting a one-site specific binding model. **C. Left.** The scheme depicts the experimental set up 2 of NanoBiT/BRET analysis of equilibrium binding between the HiBiT-FZD$_4$ and the eGFP-WNT-3A as used in the saturation binding experiments throughout this study. LgBiT and furimazine were prior to the 240 min incubation with the fluorescent probe. Created with BioRender.com. **Right.** Saturation binding of the eGFP-WNT-3A to human Nluc-FZD$_4$ was determined by the detection of NanoBRET in transiently overexpressing living HEK293A cells following 240 min incubation. Data points are presented as means ± SEM from n = 3 individual experiments, fitting a one-site specific binding model. **D.** Cell surface expression of Nluc-FZD$_4$ and HiBiT-FZD$_4$ from the two experimental setups as measured by Nanoluc or NanoBiT luminescence. Data are presented as means ± SEM from n = 3 individual experiments. Experiments were performed with eGFP-WNT-3A batch 2.

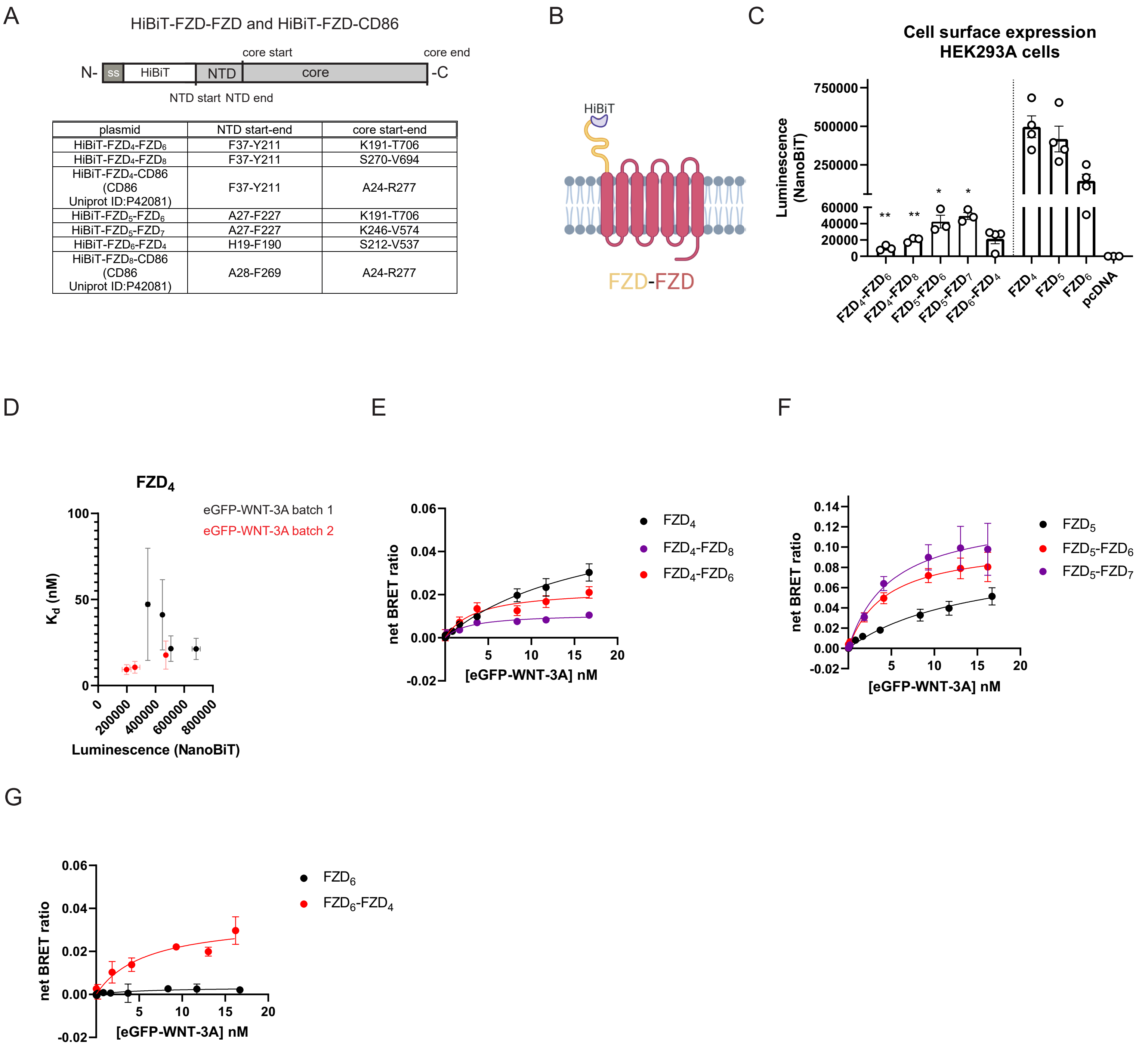

plasmid	NTD start-end	core start-end
HiBiT-FZD$_4$-FZD$_6$	F37-Y211	K191-T706
HiBiT-FZD$_4$-FZD$_8$	F37-Y211	S270-V694
HiBiT-FZD$_4$-CD86 (CD86 Uniprot ID:P42081)	F37-Y211	A24-R277
HiBiT-FZD$_5$-FZD$_6$	A27-F227	K191-T706
HiBiT-FZD$_5$-FZD$_7$	A27-F227	K246-V574
HiBiT-FZD$_6$-FZD$_4$	H19-F190	S212-V537
HiBiT-FZD$_8$-CD86 (CD86 Uniprot ID:P42081)	A28-F269	A24-R277

Fig. S4

Supplementary Figure 4. Construct validation of FZD-FZD and FZD-CD86 fusion proteins. Analysis of correlation between receptor expression levels and ligand affinity. eGFP-WNT-3A saturation binding to FZD-FZD chimeras. A. Details of HiBiT-FZD-FZD and HiBiT-FZD-CD86 constructs used to study eGFP-WNT-3A binding. First and last amino acids of FZD N-terminal domain (NTD) and FZD core or CD86 for each construct are shown. ss- signal sequence. **B.** Cartoon representation of HiBiT-FZD- constructs used to study eGFP-WNT-3A binding. Created with BioRender.com. **C.** Cell surface expression of HiBiT-tagged FZD-FZD chimeras as measured by NanoBiT luminescence (from the experiments summarised in **Figure 2B** and **4B-D**). Data are presented as means ± SEM from *n* = 3-4 individual experiments. Expression data of FZD_4, FZD_5, FZD_6 and pcDNA are also depicted in **Supplementary Figure 1**C. Statistical analysis was performed for FZD_4-FZD_6 vs FZD_4, FZD_4-FZD_8 vs FZD_4, FZD_5-FZD_6 vs FZD_5, FZD_5-FZD_7 vs FZD_5 and FZD_6-FZD_4 vs FZD_6 comparisons. **D.** Correlation between HiBiT-FZD_4 expression upon transient overexpression in HEK293A cells and corresponding K_d of eGFP-WNT-3A (batch 1 in black, batch 2 in red). Data come from individual experiments (mean ± SD) that were pooled and presented in **Figure 2B** and **Supplementary Figure 3B**. **E**. Saturation binding of eGFP-WNT-3A at FZD_4-FZD_6, FZD_4-FZD_8 and FZD_4 (data also present in **Figure 2B**) was determined by the detection of NanoBiT/BRET in transiently overexpressing living HEK293A cells following 240 min incubation. Data points are presented as means ± SEM from *n* = 3-4 individual experiments. eGFP-WNT-3A batch 1 was used in these experiments. **F.** Saturation binding of eGFP-WNT-3A at FZD_5-FZD_6, FZD_5-FZD_7 and FZD_5 (data also present in **Figure 2B**) was determined by the detection of NanoBiT/BRET in transiently overexpressing living HEK293A cells following 240 min incubation. Data points are presented as means ± SEM from *n* = 3-4 individual experiments. eGFP-WNT-3A batch 2 was used in the experiments with FZD_5-FZD_6 and FZD_5-FZD_7, and eGFP-WNT-3A batch 1 was used in the experiments with FZD_5.

G. Saturation binding of eGFP-WNT-3A at FZD_6-FZD_4 and FZD_6 (data also present in **Figure 2B**) was determined by the detection of NanoBiT/BRET in transiently overexpressing living HEK293A cells following 240 min incubation. Data points are presented as means ± SEM from n = 3-4 individual experiments. eGFP-WNT-3A batch 2 was used in the experiments with FZD_6-FZD_4, and eGFP-WNT-3A batch 1 was used in the experiments with FZD_6.

Batch 1

A

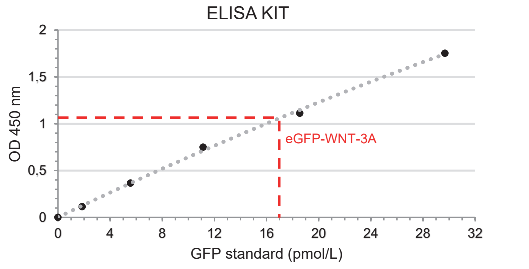

	OD 450 nm	1000x diluted	final
eGFP-WNT-3A CM	1.083	16.7 pM	**16.7 nM**

B

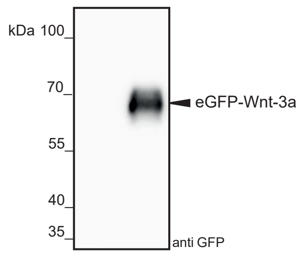

C

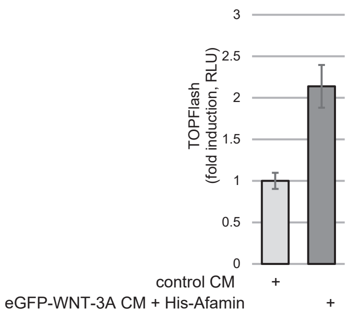

Batch 2

D

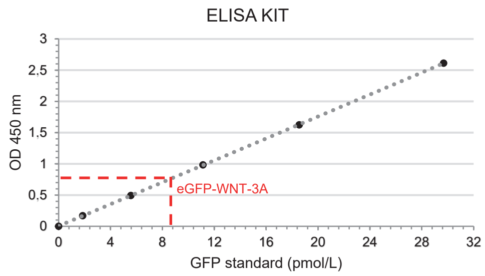

	OD 450 nm	2000x diluted	final
eGFP-WNT-3A CM	0.762	8.14 pM	**16.2 nM**

E

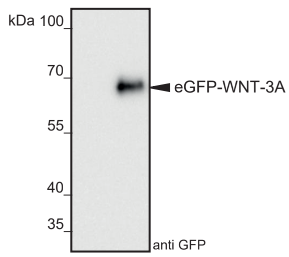

F

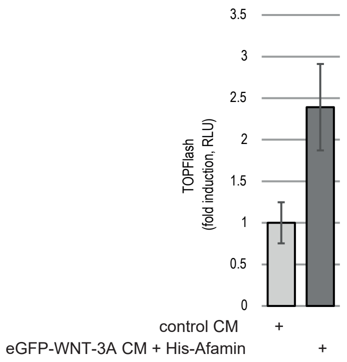

Fig. S5

Supplementary Figure 5. Validation of eGFP-WNT-3A conditioned media (CM) Batch 1 and Batch 2 used in this study. **A**, **D**. GFP ELISA assay (GFP ELISA® kit, Abcam, ab171581) to determine the concentration of eGFP-WNT-3A present in each CM preparation. **B**, **E**. Western blotting (anti GFP, Santa Cruz Biotechnology, sc-9996) confirming presence of soluble full length eGFP-WNT-3A in the CM. **C**, **F**. TOPFlash TCF reporter assay showing activity of the indicated CM in HEK293T cells.

References:

[1] Arthofer, E., Hot, B., Petersen, J., Strakova, K., Jager, S., Grundmann, M., Kostenis, E., Gutkind, J. S., and Schulte, G. (2016) WNT Stimulation Dissociates a Frizzled 4 Inactive-State Complex with Galpha12/13, *Mol Pharmacol 90*, 447-459.

[2] Hot, B., Valnohova, J., Arthofer, E., Simon, K., Shin, J., Uhlen, M., Kostenis, E., Mulder, J., and Schulte, G. (2017) FZD10-Galpha13 signalling axis points to a role of FZD10 in CNS angiogenesis, *Cell Signal 32*, 93-103.
